# COVID-19 and Herpes Simplex Virus Infection: A Cross-Sectional Study

**DOI:** 10.7759/cureus.18022

**Published:** 2021-09-16

**Authors:** Mohammed Shanshal, Hayder Saad Ahmed

**Affiliations:** 1 Dermatology, Basildon University Hospital, Basildon, GBR; 2 Department of Dermatology and Venereology, University of Tikrit, College of Medicine, Tikrit, IRQ

**Keywords:** covid-19-related immune dysregulation, coronaviridae, dermatologic manifestations of covid-19, herpes simplex infection, covid-19

## Abstract

Background

Despite being variable and poorly characterized, the reported cutaneous manifestations of coronavirus disease 2019 (COVID-19) are of increasing concern.

Methodology

This study aimed to determine the prevalence and possible association between COVID-19 and herpes simplex virus (HSV) infection. A nine-item questionnaire was sent to 120 polymerase chain reaction-confirmed COVID-19 patients with a response rate of 66.67%. This cross-sectional observational study included 80 patients with mild-to-moderate COVID-19 infection who did not require hospitalization or steroid therapy.

Results

One or more HSV infections were observed in 28 patients (35%) with COVID-19 infection, including 10 (35.7%) males and 18 (64.29%) females. Of the 28 patients, fever was reported in 17 (75%) during COVID-19. Most of the respondents (78%) described a single HSV reactivation, 14.29% had two attacks, and 7.14% experienced three attacks. Compared to previous non-COVID-19-related HSV reactivation, the COVID-19-related attacks were more severe in 12 (42.85%) patients, equally severe in five (17.85%) patients, and less severe in one (3.57%) patient. Interestingly, 10 (35.71%) patients developed an initial symptomatic HSV attack during COVID-19 infection.

Conclusions

This study demonstrated a possible association between COVID-19 infection and primary HSV infection or reactivation. COVID-19 direct neuronal effect in addition to COVID-19-related psychological stress, fever, and immunological dysregulation could play a potential role in HSV reactivation or primary infection during COVID-19.

## Introduction

For about a year now, the world has been under the grip of the coronavirus disease 2019 (COVID-19) pandemic. More than 183 million cases and approximately four million deaths have been reported globally [1]. COVID-19 is caused by severe acute respiratory syndrome coronavirus 2 (SARS-CoV-2), a member of the Coronaviridae family. In the 21st century, two other major respiratory disease outbreaks were caused by closely related coronaviruses, including the severe acute respiratory syndrome and the Middle East respiratory syndrome [2].

Although respiratory manifestations seem to be predominant, an increasing number of COVID-19-related cutaneous manifestations have been reported, including maculopapular, urticarial, purpuric, chilblain-like, and vesicular eruptions. Other less commonly reported lesions include livedo reticularis-like, erythema multiforme-like, papulosquamous, necrotic lesions, and gangrene [3-5].

Herpes simplex virus (HSV), an enveloped DNA virus belonging to the Herpesviridae family, is a ubiquitous pathogen that commonly infects humans. It has been estimated that two-thirds of the population under 50 years of age are infected with HSV-1 [6]. After primary infection, HSV can establish a life-long latent infection that can occur in the sensory ganglia via retrograde transport through peripheral neurons. The virus can be reactivated periodically in response to a variety of stimuli, including psychological stress, fever, sunlight, hormonal imbalance, immunosuppression, and surgical resection [7,8]. When HSV-1 infection is symptomatic, the most common clinical manifestations are gingivostomatitis and pharyngitis in children and adults, respectively, whereas herpes labialis “cold sore” represents the most frequent sign of HSV-1 reactivation [9,10]. Other less frequent cutaneous manifestations of HSV-1 include herpetic whitlow, erythema multiform, eczema herpeticum, and herpes gladiatorum [11-14]. A preprint of this article was previously submitted to medRxiv.

## Materials and methods

A cross-sectional observational study was conducted at Baghdad Teaching Hospital, where data were obtained from an online survey conducted between August and October 2020. Ethical approval for this study was obtained from the Arab Board of Health Specialization Committee. Eligibility criteria were as follows: any age, polymerase chain reaction (PCR)-confirmed mild-to-moderate COVID-19 infection, not admitted to the respiratory care unit, or required steroid therapy. All patients who were suspected of having COVID-19 infection but did not have a confirmatory PCR, had severe COVID-19 infection, or were taking immunosuppressive drugs were excluded from the study. A nine-item online questionnaire was created and sent to 120 patients with PCR-confirmed mild-to-moderate COVID-19. The survey included a range of measures to examine the prevalence, severity, and timing of herpes simplex during COVID-19 infection. Participants reported their age, sex, COVID-19 status, history and severity of past HSV infection, history and severity of HSV during COVID-19, history of fever, and the timing of HSV infection during COVID-19. The clinical features of herpes labialis were used to make the diagnosis, which included classic grouped lesions (papules, vesicles, ulcers) on the lip accompanied by pain, stinging, and/or discomfort.

## Results

The questionnaire was completed by 80 out of 120 patients who received an online survey with a response rate of 66.67%. The mean age and SD were 33.87 ± 9.46 years. A total of 28 (35%) patients reported single or multiple episodes of HSV reactivation (Table 1).

**Table 1:** Frequency distribution of COVID-19-related HSV infection, fever, and previous HSV infection among included patients. COVID-19: coronavirus disease 2019; HSV: herpes simplex virus

COVID-19 and herpes simplex infection	Yes	No
Fever	21 (75%)	7 (25%)
COVID-19-induced herpes simplex infection	28 (35%)	52 (65%)
Positive history of recurrent herpes	18 (64.29%)	10 (35.71%)

**Figure 1:**
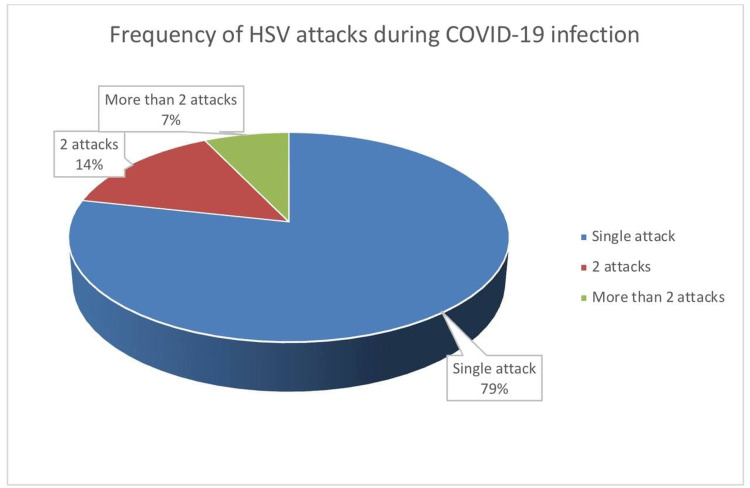
Frequency of HSV infection among COVID-19 patients. COVID-19: coronavirus disease 2019; HSV: herpes simplex virus

The majority of patients (n = 24, 78.57%) reported a single HSV reactivation during their COVID-19 infection, while 14.29% and 7.14% experienced two or more attacks, respectively (Figure 1).

**Figure 2:**
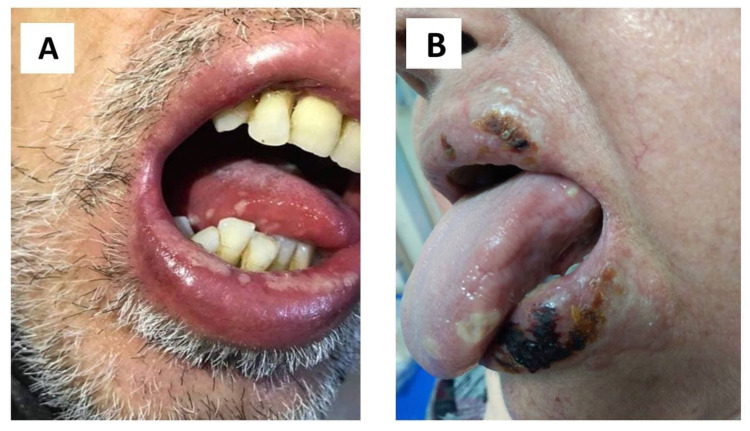
Two examples of HSV infection (herpes labialis) in COVID-19 patients. COVID-19: coronavirus disease 2019; HSV: herpes simplex virus

Of the 28 patients, 18 (46.29%) reported recurrent attacks of HSV reactivation before COVID-19 infection. A total of 28 (35%) patients with PCR-confirmed COVID-19 reported single or multiple attacks of HSV infection (Figure 2), including 18 (64.29%) females and 10 (35.71%) males, with a mean age of 38.4 years and SD of 13.64.

Compared to previous attacks, COVID-19-related HSV reactivation was more severe, equally severe, or less severe in 42.86%, 17.68%, and 3.57%, respectively. The onset of HSV infection varies among individuals after the onset of COVID-19. The mean time of onset of the first HSV infection was 8 ± 6.48 days while the onset on the second and third attacks were 11 ± 3.44 and 22 ± 3.53 days, respectively (Table 2).

**Table 2:** The mean time of HSV onset after acquiring COVID-19. COVID-19: coronavirus disease 2019; HSV: herpes simplex virus

Timing of HSV reactivation	No.	Mean day of HSV onset	SD
First attack	28	8	6.48
Second attack	6	11	3.44
Third attack	2	22	3.53

## Discussion

Depending on the host cell type, HSV can produce lytic and latent lytic stages, where the lytic stage can occur in a variety of tissue types, while the latent stage tends to occur in neuronal tissues [15]. The lytic stage can result from viral replication in the epithelial cells of the oral and genital mucosa, eye, or skin producing labial herpes (cold sores), genital herpes, herpetic keratitis, and herpetic whitlow or eczema herpeticum, respectively. HSV is a neurotropic virus that can establish latency in the neuronal dendrites of the sensory ganglia that supply primary epithelial tissues [16]. The immune system plays a vital role in controlling HSV replication, driving the virus into a latent state for prolonged periods [17].

The number of females surpassed that of males as the rate of HSV seropositivity is slightly higher in females aged between 15 and 39 years than in males [18,19]. Our findings suggest that reactivation of HSV is frequent in patients with COVID-19, and could serve as a clue to COVID-19 infection in patients with respiratory symptoms.

Four pathomechanisms have been proposed to explain the higher frequency of herpes reactivations during COVID-19. The first is the COVID-19-related immune dysregulation. COVID19 is associated with unique immune dysregulation, including CD4 cell and NK cell cytopenias and sustained inflammatory interleukin stimulation such as interleukin-6 (IL-6) and tumor necrosis factor-α [20]. Immune dysregulation causes HSV-1 to burst from latency and travel anterogradely to epithelial surfaces, where viral replication and lytic stages occur [21]. Interestingly, a potential relationship between the cytokine IL-6, which is overrepresented in COVID-19 patients [22], and reactivation of HSV has been proposed [23]. Compared to mice receiving control antibodies, HSV-latently infected mice injected with neutralizing anti-IL-6 antibodies show a lower frequency of virus reactivation [24].

The second is the direct effect of COVID-19 on the neurons. COVID-19 has a potential neurotropic mechanism that explains the neurological manifestations associated with COVID-19 such as loss of taste and smell, headache, dizziness, meningitis, cerebrovascular disease, and acute Guillain-Barré syndrome. SARS-CoV-2 has an affinity to bind to the angiotensin-converting enzyme 2 (ACE2) receptor; hence, cells that express ACE2 such as neurons and glial cells are vulnerable to SARS-CoV-2 infection [25].

The third is the COVID-19-related psychological stress. COVID-19 is associated with significant psychological and physical stress. It has been postulated that stress impairs the cytotoxic T-cell surveillance of latently infected neurons, resulting in the replication and activation of latent viruses [26]. Other hypotheses include stress-related relapse of catecholamines and glucocorticoids (stress hormones) and their direct and indirect effects on HSV reactivation [27].

**Figure 3:**
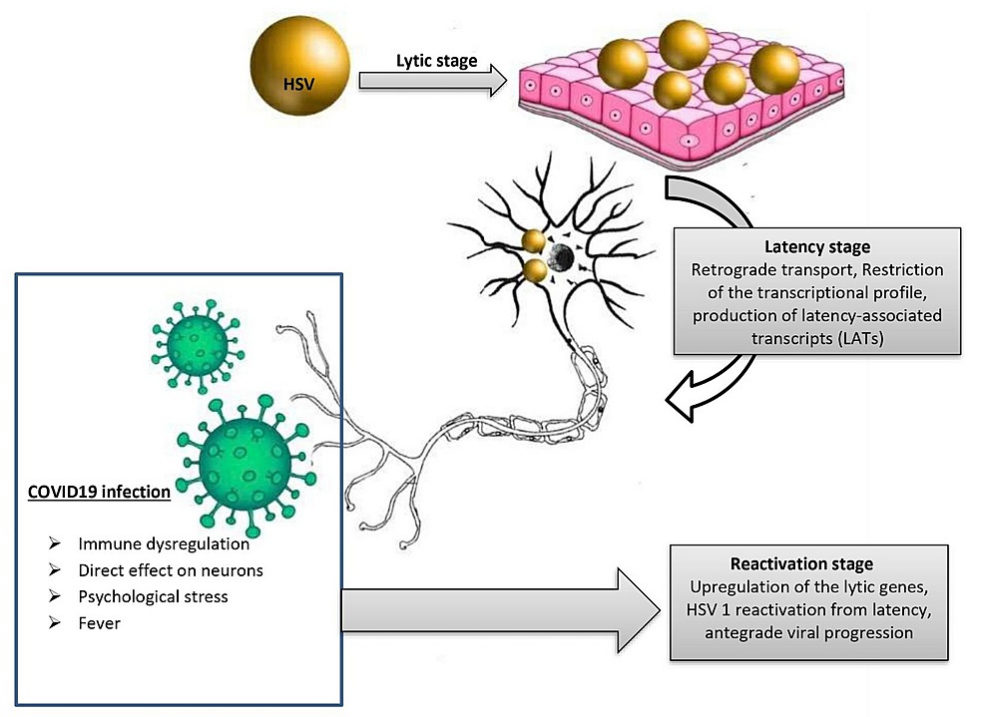
Proposed pathomechanisms of HSV reactivation during COVID-19 infection. After primary infection, HSV can remain in the latent stage for years. COVID-19-related immune dysregulation, fever, stress, and the direct neuronal effect of the virus can result in the upregulation of HSV lytic genes, reactivation of the latent virus, and viral replication again. COVID-19: coronavirus disease 2019; HSV: herpes simplex virus

The fourth is COVID-19-related fever. Seventy-five percent of patients with HSV in the survey reported fever. Fever is a strongly linked environmental trigger of human HSV reactivation either through a direct effect on latently infected neurons and/or the secretion of pyrogenic cytokines including IL-6 [28]. All four pathomechanisms are illustrated in Figure 3.

Le Balc’h et al. suggested that COVID-19 infection could be a risk factor for Herpesviridae reactivation and subsequent pulmonary infection in patients with COVID-19 severe acute respiratory distress syndrome [29].

Elsaie et al. described two cases of varicella-zoster virus (VZV, another member of the Herpesviridae family) in patients with COVID-19 infection. After primary infection, VZV remains dormant in neuronal tissues and is reactivated in a similar fashion to HSV [30]. HSV diagnosis was done clinically and by the patients themselves. Recall bias is also a limitation of this study; hence, more prospective or retrospective case-control studies are recommended.

## Conclusions

Although it is primarily a respiratory virus, COVID-19 can cause a variety of mucocutaneous manifestations in people of all age groups. COVID-19 infection can trigger reactivation of the latent HSV by upregulating the expression of lytic genes and supporting the antegrade progression of the activated viruses toward the epithelial tissues. COVID-19-related immune dysregulation, psychological stress, fever, and direct neuronal effects play a role in the activation of different cellular processes that result in increased HSV lytic gene expression and reactivation of the virus. Herpes simplex reactivation in patients with new respiratory symptoms could serve as a new clue for the emerging COVID-19 infections.
